# Detection of Pathogenic Serogroups and Virulence Genes in *Listeria monocytogenes* Strains Isolated from Beef and Beef Products Retailed in Gauteng Province, South Africa, Using Phenotypic and Polymerase Chain Reaction (PCR)-Based Methods

**DOI:** 10.1155/2024/8891963

**Published:** 2024-03-13

**Authors:** James Gana, Nomakorinte Gcebe, Rebone Moerane, Yusuf B. Ngoshe, Khomotso Moabelo, Abiodun A. Adesiyun

**Affiliations:** ^1^Department of Production Animal Studies, Faculty of Veterinary Science, University of Pretoria, Private Bag X04, Onderstepoort, Pretoria 0110, South Africa; ^2^Department of Agricultural Education, School of Vocational Education, Federal College of Education, P.M.B. 39, Kontagora, Niger, Nigeria; ^3^Bacteriology Department, Onderstepoort Veterinary Research, Agricultural Research Council, Pretoria, South Africa; ^4^School of Veterinary Medicine, Faculty of Medical Sciences, University of the West Indies, St. Augustine, Trinidad and Tobago

## Abstract

South Africa recently (2017-18) experienced the largest outbreak of human listeriosis in the world caused by *L. monocytogenes* following the consumption of “polony,” a ready-to-eat meat product. Most (59%) cases originated from Gauteng province, South Africa. As a follow-up study to the outbreak, we used standard bacteriological and molecular methods to determine the prevalence of pathogenic and virulent serogroups of *L. monocytogenes* in various beef and beef products retailed in Gauteng province, South Africa. The overall prevalence of *Listeria* spp. was 28% (112/400), comprising *Listeria monocytogenes* (9.3%), *Listeria innocua* (16.3%), and *Listeria welshimeri* (2.5%) (*p* < 0.001). It is crucial to have detected that the region (*p*=0.036), type of product (*p*=0.032), and temperature at storage (*p*=0.011) significantly affected the occurrence of *L. monocytogenes* in beef products. It is alarming that pathogenic serogroups 4b-4d-4e (51.4%) and 1/2a-3a (43.2%) were detected among the isolates of *L. monocytogenes*. Importantly, they were all carriers of seven virulence-associated genes (*hlyA, inlB, plcA, iap, inlA, inlC, and inlJ*). Our study also demonstrated that 16.7% of “polony” samples investigated were contaminated with *L. monocytogenes.* Considering that pathogenic and virulent *L. monocytogenes* contaminated beef and beef products retailed in South Africa, the food safety risk posed to consumers remains and cannot be ignored. Therefore, it is imperative to reduce the contamination of these products with *L. monocytogenes* during beef production, processing, and retailing to avoid future outbreaks of human listeriosis in the country.

## 1. Introduction

Listeriosis is a well-known food-borne infection mainly affecting humans, particularly immunocompromised, pregnant, young, and old individuals [1]. *Listeria monocytogenes* is the leading cause of human and animal listeriosis, posing the highest risk to food safety and public health and responsible for various clinical manifestations [2]. *L. innocua* has been generally considered nonpathogenic, and human listeriosis infections due to *L. innocua* constitute rare occasions. However, few reports of the implication of *L. innocua* in human listeriosis have been documented [3]. *L. innocua* is also known to share the same food environment with pathogenic *L. monocytogenes,* with the potential for the transfer of virulence and resistance genes between both species of *Listeria* [4]. Several global human listeriosis outbreaks have been reviewed [5]. *L. monocytogenes* has been isolated from several types of foods that serve as vehicles for transmitting the pathogen to humans and causing listeriosis globally [6]. The types of food implicated include milk and milk products [7], vegetables [8], and meat and meat products [9].

The serogroups and serotypes of *L. monocytogenes* commonly detected in foods, particularly in beef and beef products, include 4b-4d-4e and 1/2a-3a,1/2b-3b-7 [10], 1/2b, 4b, 1/2a, 3b, 4d, 4e [11], are of epidemiological and public health relevance because they are associated with human listeriosis [12].

Several virulence genes have been documented in *L. monocytogenes strains recovered from beef and beef products and other food sources* [13, 14]. The frequency of virulence genes is *higher in L. monocytogenes* than in other *Listeria* spp [15, 16] and varies with the geographical location, source, and types of samples [10]. The virulence genes possessed by strains of *L. monocytogenes* have been demonstrated to be responsible for the organism's pathogenicity [17, 18], especially those in the *Listeria* Pathogenicity Islands (LIPIs) [17–19]. The genes perform different roles in the pathogenicity of *L. monocytogenes,* including facilitating the infectious life cycle and survival in the food processing environment by the virulence genes in the LIPI-1 and LIPI-3 clusters [20]. For example, *inlA*, *inlB*, and *inlC* play a role in the adherence to, and internalization by the host cell, virulence genes *hly*, *plcA*, and *plcB* are responsible for escape from the vacuoles, *htp* enables intracellular replication, and *actA* gene facilitates cellular movement by *L. monocytogenes* [17, 18, 21].

Between 2017 and 2018, South Africa experienced the world's largest outbreak of human listeriosis [22]. Whole genome sequencing (WGS) identified *L. monocytogenes* sequence type 6, which originated from “polony,” an RTE beef product, as the outbreak's origin [23]. Matle et al. [10] reported the prevalence of *L. monocytogenes* to be 10.1%, 13.5%, and 19.5% for raw intact meat, RTE meat products, and processed meats in South Africa. There is, however, a shortage of current information on epidemiological data on the samples assessed for contamination with *L. monocytogenes*, the risk posed by RTE foods, and the species of *Listeria* other than *L. monocytogenes* in beef and beef products.

Of relevance to the current study conducted in Gauteng province, one of the nine provinces in South Africa, is that 57.93% of the country-wide confirmed 1060 cases in the recent human listeriosis outbreak the country originated from the province [22, 24]. The current study, therefore, investigated the occurrence of pathogenic serogroups and virulence genes in *L. monocytogenes* and other species of *Listeria* in beef and beef products using phenotypic and polymerase chain reaction (PCR)-based methods. The study characterized the isolates of *L. monocytogenes* regarding their serogroups and carriage of virulence genes. The study also investigated the effects of selected variables on detecting *Listeria* spp.

## 2. Materials and Methods

### 2.1. Physicochemical Properties and Microbiological Properties of Biltong and Polony

A total of five categories of beef and beef products (beef burger, ““biltong”, minced beef, brisket, cold beef (“polony” and Vienna) were sampled in the current study, but only two (“Biltong” and “Polony”) are unique, and popularly consumed in South Africa.

#### 2.1.1. “Biltong”

Biltong is an RTE beef product popularly consumed across South Africa. It is produced in dry and moist forms for consumers at sale outlets. Biltong is made from thin slices of raw muscle meat that are marinated or cured (spices and organic acids), refrigerated, and dried (air or oven) without cooking or thermal lethality step [25, 26]. The production of “Biltong” is a relatively straightforward process, increasing traditional home production and small-scale production on farms and butcheries and modern manufacturing methods in the country with no established content and microbial quality control. It is a product like the American dried meat product known as Jerky [27], which, unlike the South African “Biltong,” is subjected to thermal lethality step treatment before drying. Dry “biltong” in South Africa is characterized by a low water activity (*a*_*w*_) ranging from 0.65 to 0.68 [28]. It has been documented that the critical *a*_*w*_ in which dry “biltong” is microbiologically stable is below 0.68 [29]. On the other hand, moist “biltong” has higher moisture content, ranging from 0.85 to 0.89 [30]. The pH ranges of “biltong” in South Africa have been variable. Petit et al. [28] reported that the pH values of “biltong” varied from 5.00 to 6.26, with an average of 5.35 and 5.58 for dry and moist “biltong,” respectively. Reports by others documented some “biltong” pH ranges between 4.81 and 5.83 [29, 31, 32]. Gavai et al. [33], using the standard process for processing “biltong” assessed the effect of the drying process on the population of inoculated *L. monocytogenes* and reported that an internal water activity (*a*_*w*_) reached <0.85 at 5-log reduction levels and ensured that conditions were lower than that which would support bacterial growth, including *L. monocytogenes.* To date, “biltong” (moist or dry) has not been associated with human listeriosis.

Only dry biltong was sampled and processed in the current study.

#### 2.1.2. “Polony”

“Polony,” a bologna sausage, is a low-cost, readily available food popular across all socioeconomic groups in urban and rural communities in South Africa. It is produced mainly from mechanically recovered meat (beef/pork/chicken) and processed by food manufacturers in South Africa [22]. The extensive use of meat trimmings (beef, pork, or chicken) makes “polony,” an inexpensive and affordable meat product [34]. The pH ranges of “biltong” in South Africa have been variable. The product is typically sliced and served cold. “Polony” has been documented to have a shelf life of five months and is produced in large quantities by several manufacturers for local consumption and export [23]. “Polony” elicited considerable anxiety in the South African population with the implication that the RTE product, which originated from a single facility in the country, was demonstrated to be responsible for the large outbreak of human listeriosis. An average pH of 5.35 and 5.58 for dry and moist polony, respectively, has been reported for “polony” [35]. Kivikari [36] reported that the pH and buffering capacity of the raw materials used in cooked sausage or “polony” affected the final pH.

### 2.2. Study Design

The cross-sectional study was conducted at 48 retail outlets in Gauteng province, South Africa. These were selected based on information from the Consumer Goods Council of South Africa (CGCSA) (https://www.cgcsa.co.za/). The number of samples collected from each type of outlet was proportional to its size and availability during sampling visits using a convenience sampling approach. The sample size for the study was determined using the formula by Thrusfield [37],

(1) $$\begin{array}{c} n_{o}=\left\{\frac{1.96^{2}\;xP_{\exp}\;x\;\left(1\;–\;P_{\exp}\right)}{d^{2}}\right\}, \end{array}$$

where *P*_exp_ is the expected prevalence and *d* is the desired precision

A *P*_exp_ value of 14.7% [10] and a *d* value of 3.5%,

(2) $$\begin{array}{c} n_{o}=\frac{3.84\;x\;0.147\;x\;0.853}{0.0012}=\frac{0.4815}{0.0012}=401,\\ n_{o}=401\;\text{samples}. \end{array}$$

For the study, a total of 400 samples were collected from retail outlets across Gauteng province, South Africa, and distributed across 48 retail outlets, as shown in Supplementary Table S1. Overall, from the 48 outlets, 8 samples were collected from 32 outlets (*n* = 256), while 9 samples were obtained from 16 outlets (*n* = 144).

The samples were collected between October 2019 and April 2021 during a single visit to each outlet, including raw, chilled, and frozen beef and dried beef-based RTE products.

### 2.3. Sample Collection, Isolation, and Identification of *Listeria* spp

For the 48 outlets from where samples were collected, at 32 (66.7%) outlets, 8 samples were collected per outlet, while at 16 (33.3%), 9 samples were obtained per outlet. The types of samples collected per outlet were based on the beef and beef products available during the visit. The strategy for collection and the number of samples collected from each outlet are shown in Supplementary data, Table S2. The maximum number of samples collected from the four categories of the outlets was 12 (*n* = 160), 8–10 (*n* = 128), 4–6 (*n* = 80), and 1-2 (*n* = 32) for chain, large, medium, and small retail outlets, respectively. A total of 13 types of raw and beef products (beef steak, liver, tripe, chunk, sausage, raw beef, beef “polony,” Russian “polony,” Vienna, dry “biltong,” moist “biltong,” beef patties, and beef burger) were sampled from the retail outlets (Supplementary Table S2).

Matle et al. [10] described isolating and confirming suspect *Listeria* spp. using phenotypic assays. For the study, the validated *Listeria* Precis method, according to a protocol by Thermofisher Scientific and reported by Matle et al. [10], was used with some modifications. Ten grams of each sample was aseptically transferred into a stomacher bag containing 225 mL of ONE broth-*Listeria* (Oxoid, Basingstoke, UK) for selective enrichment. The samples were homogenized in a Stomacher (Stomacher Lab Blender 400, Seward Ltd., West Sussex, UK) for 5 min at 15,493 × g speed, followed by aerobic incubation at 35°C for 24 h for enrichment. Thereafter, for isolation of *Listeria*, 10 *μ*L of selective enriched broth sample was inoculated onto Brilliance-*Listeria* agar (BLA) plates (Oxoid, Basingstoke, UK) and incubated at 35°C for 24 h. Based on the phenotypic appearance of the colonies on BLA, which presumptively classified green-blue colonies without a halo as *Listeria* spp. Blue colonies with white/cream halo were tentatively identified phenotypically as *L. monocytogenes*. Single colonies of suspected *Listeria* spp. (colonies that appeared blue without a halo) and *L. monocytogenes* (blue colonies with a white/cream halo) were subcultured on BLA for further purification [38].

### 2.4. Molecular Confirmation of *Listeria*

#### 2.4.1. Extraction of DNA from Selective Enriched Broth Cultures and Colonies

DNA was extracted by heating method, and the crude extract was used in subsequent PCR protocols [39]. Briefly, 2 ml aliquots of samples in selective enrichment broth were spun at 15,493 x g for 5 minutes in a microcentrifuge (Eppendorf, South Africa). The pellets were suspended in 200 *μ*L of sterile distilled water, heated to 95°C in a dry block for 10 minutes, cooled at room temperature for 5 minutes, and centrifuged at 15,493 × g for 5 minutes. The supernatant was pipetted into sterile tubes, and the pellet was discarded. The DNA in the supernatant was used as template DNA in PCR assays.

#### 2.4.2. Determination of the Species of *Listeria* Using mPCR

The multiplex PCR was used to determine the five species of the *Listeria* isolates (*L. grayi, L, innocua, L. ivanovii, L. monocytogenes, L. seeligeri,* and *L. welshimeri*) as described by Ryu et al. [40]. The multiplex PCR mix was prepared as follows: 12.5 *μ*L of 2x red Taq master mix, 5 *μ*L (Lasec, SA, Pty, Cape Town, South Africa) nuclease-free water, 5 *μ*L DNA template, and 4 *μ*L of 20 *μ*M primer mix for PCR assay. Multiplex PCR was performed with an initial denaturation at 94°C for 5 min, 35 cycles of denaturation at 94 C for 30 s, annealing at 60°C for 30 s, and extension at 72°C for 30 s, and final extension at 72°C for 5 min. PCR amplicons were electrophoresed on a 3.0% agarose gel using 1 × Tris-acetate-EDTA (TAE) buffer and stained with ethidium bromide. *L. monocytogenes* ATCC 19111, *Listeria innocua* ATCC 33090, *L*. w*elshimeri* ATCC 35897, *L. grayi* ATCC 25401, *L. ivanovii* ATCC 19119, and *L. seeligeri* ATCC 35967 were used as positive controls, *Campylobacter fetus* ATCC 27373 as a negative control, and water as a blank. The primer sequences used to determine the species of *Listeria* in the study are listed in Supplementary data, Table S3.

#### 2.4.3. Determination of the Serogroups of *L. monocytogenes* Isolates

Multiplex PCR was used to classify *L. monocytogenes* strains into serogroups by targeting genes (*ORF2110, ORF2819, Imo1118, Imo0737, and prs*) as described by Doumith et al. [41]. The *prs* gene was used as a target marker for the *Listeria* species. PCR mix was prepared as follows: 12.5 *μ*L of 2x of red Taq master mix (Lasec, South Africa), 5 *μ*L nuclease-free water, 5 *μ*L DNA template, and 4 *μ*L of 20 *μ*M primer mix. The PCR cycling conditions were as follows: initial denaturation step at 94°C for 3 min; 35 cycles of 94°C for 0.40 min at 53°C for 1.15 min and 72°C for 1.15 min, and one final cycle at 72°C for 7 min and amplification was done in a Thermocycler. The PCR products were subjected to electrophoresis on 3% ethidium bromide-stained agarose gel for 3 h at 100 v. *L. monocytogenes* ATCC 19111 was used as a positive control, *Campylobacter fetus* ATCC 27373 was used as a negative control, and water was used as a blank. The primer sequences used to determine the serogroups of *L. monocytogenes* in the study are listed in Supplementary data, Table S4.

#### 2.4.4. Detection of Virulence Genes in *L. monocytogenes* Isolates

Multiplex PCR, earlier described by Rawool et al. [42], was used to identify the *hlyA, plcA*, *actA*, and *iap* virulence genes specific to *L. monocytogenes*. The second multiplex PCR was used to determine the extra virulence genes *inlA*, *inlB*, *inlC*, and *inlJ* [43]. PCR mix was prepared as follows: 12.5 *μ*L 2× DreamTaq master mix, 5 *μ*L nuclease-free water, 5 *μ*L template DNA, 3 *μ*L primer mix. The PCR cycling conditions were as follows: The initial denaturation step at 2 min at 94°C, 35 cycles of 94°C for 30 s, 55°C for 30 s, 72°C for 1 min, and a final extension at 72°C for 10 min. The PCR products were electrophoresed on a 3% agarose gel, and results were captured using a gel documentation system (Vacutec, SA). The primer sequences used to detect virulence genes are listed in Supplementary data, Table S5.

### 2.5. Data Analysis

Laboratory data generated for the prevalence of the six species of *Listeria,* serogroups, and virulence genes were analyzed using Stata software (StataCorp LLC, College Station, Texas, USA), and the association of variables with the detection of *Listeria* or selected characteristics was determined using Fisher's Exact and Chi-square tests. The significant difference was evaluated using (*P* <  0.05), and percentages were calculated at a 95% confidence interval. A descriptive analysis was also performed using StataCorp to assess the prevalence of the six species by the locations of the outlets, type of retail outlets, type of beef and beef products, storage temperature, and product status.

## 3. Results

Of the 400 samples of beef and beef products, the prevalence of the *Listeria* genus was 28.0% (112/400). The prevalence of the six species of *Listeria* investigated was 16.3%, 9.3%, 2.5%, 0%, 0%, and 0% for *L. innocua*, *L. monocytogenes*, *L*. *welshimeri, L. grayi, L. ivanovii,* and *L. seeligeri,* respectively (*P* < 0.05).

From the 48 outlets, regardless of the type of beef and beef products tested, 26 (54.1%) yielded *L. monocytogenes*, with the positivity rate ranging from 22.2% (2/9) to 33.3% (3/9). Samples from 36 (75%) of 48 outlets were positive for *L. innocua*, with the range of frequency of detection in positive outlets being from 22.2% (2/9) to 44.4% (4/9). Only 8 (16.7%) of the outlets yielded *L. welshimeri,* ranging from 22.2% (2/9) to 25% (2/8) in positive outlets.

For the selected RTE foods, the respective prevalence of *L. monocytogenes*, *L. innocua*, and *L. welshimeri* was 16.7% (5/30), 16.7% (5/30) and 0% (0/30) for beef “polony” (*P* > 0.05); 5.7% (2/35), 14.2% (5/35) and 0% (0/35) for beef Vienna (*P* > 0.05); and 3.6%, 12.7% and 1.8% for “biltong” (*P*=0.037).

The prevalence of *L. monocytogenes, L. innocua,* and *L. welshimeri* in beef and beef products and the univariate analyses of associated factors is shown in Table 1.

The prevalence of *L. monocytogenes* was 9.3% (37/400), and statistically significant differences were detected for three variables, namely, the retail outlets' regional locations (*P*=0.036), the type of product (*P*=0.032), and the temperature of storage (*P*=0.011).

The prevalence of *L. innocua* was 16.3% (65/400). The temperatures at which the beef and beef products were kept pre-sale had a statistically significant (*P* < 0.001) effect on the prevalence of *L. innocua*.

*L. welshineri* contaminated 2.5% (10/400) of the samples. None of the variables had a statistically significant (*P* > 0.05) effect on the prevalence of *L. welshimeri.*

From the total of 48 retail outlets from where samples were collected, *L. monocytogenes* was detected in 25 (52.1%) at a frequency range of 11.1% (1/9) to 33.3% (3/9); *L. innocua* was found in 36 (75%) outlets at a frequency range from 11.1% (1/9) to 37.5% (3/8); *L. welshimeri* was isolated from only 8 (16.7%) outlets with the isolation frequency lowest at 11.1% (1/9) and the highest being 25% (2/4) as displayed in Supplementary data, Table S1.

Overall, the frequency of distribution of the serogroups among 37 isolates of *L. monocytogenes* was 43.2% (16/37), 5.4% (2/37), and 51.4% (19/37) for serogroup 1/2a-3a, 1/2c-3c, and 4b-4 d-4e, respectively (*P* < 0.001). The distribution of the serogroups by region, type of retail outlets, kind of beef and beef products, storage temperature, and status of the product is shown in Table 2. Statistically significant differences were detected only in the frequency of serogroups by region for 4b-4d-4e (*P*=0.023) and for the type of outlet for 1/2a-3a (*P*=0.003). The detection frequency of the eight virulence genes was 100% (37/37) each for seven virulence genes (*hlyA, inlB, plcA, iap, inlA, inlC,* and *inlJ*) but 97.3% for virulence gene *actA*. The frequency of detecting *actA* across the three variables was 90% (regions) to 93.8% (type of product).

Seven virulence genes were detected in the three serogroups (1/2a-3a, 1/2c-3c, and 4b-4d-4e), but the *actA* gene was detected in 93.8% (15/16) of the isolates in serogroup 1/2a-3a.

## 4. Discussion

Our findings that *L. monocytogenes* contaminated 9.3% of the beef and beef products tested have food safety implications for consumers because *L. monocytogenes*, the most important species of *Listeria,* is frequently associated with cases and outbreaks of human listeriosis [44]. A similar prevalence of 8.3% for *L. monocytogenes* was reported for beef and beef products (raw beef, RTE, milled beef, offal, and organs) sampled at retail outlets in Mpumalanga province, South Africa [45]. However, Matle et al. [10] reported a higher pathogen prevalence (14.7%) in the country's meat products. The differences in the findings between both studies that used the same detection methods may be due to several factors. These factors include that the current study was conducted on beef and beef products sampled from retail outlets in Gauteng province. In contrast, the study reported by Matle et al. [10] was done on meat and meat products (poultry, cattle, sheep, pork, and game); meat samples collected from the three major ports of the country and abattoirs, meat processing plants, butcheries, and retail outlets. This is because the sources of the meat products can potentially influence the prevalence of *L. monocytogenes*. It has been documented that all these variables potentially affect the prevalence of *L. monocytogenes* in meat [46].

The study also documented, for the first time in Gauteng province, South Africa, the prevalence of three species of *Listeria* (*L. monocytogenes*, *L. innocua,* and *L. welshimeri)* in beef and beef products according to retail outlet location, types of outlets, and beef products, and the virulence/pathogenic characteristics of the *L. monocytogenes* isolates.

The prevalence of *L. innocua* (16.3%) in the present study is lower than the 21.3% in RTE food samples in Johannesburg, South Africa [47]. The organism has been recovered at varying frequencies in meat products elsewhere, such as in Spain, 13.9% [48], and China, 28.9% [49]. It is well established that, unlike *L. monocytogenes*, the most important *Listeria* species implicated in human listeriosis, *L. innocua* is considered nonpathogenic [50]. Rare reports associating *L. innocua* with human listeriosis have been documented in immunocompromised individuals [3]. The detection of *L. innocua* in beef and beef products in the current study may, therefore, not have clinical significance for human listeriosis, but they are known to share the same food niche or environment with *L. monocytogenes* [48], with the possibility of transfer of genes (e.g., virulence and resistance genes) between *L. monocytogenes* and *L. innocua.* Furthermore, WGS confirmed the presence of the *L. monocytogenes* pathogenicity island (LIPI) in strains of *L. innocua*. [51]. These findings suggest that the pathogenic potential of *L. innocua* in humans cannot be ignored.

In our study, the prevalence (2.5%) of *L. welshimeri*, considered nonpathogenic, is the first documentation of this *Listeria* species in beef and beef products in Gauteng province. Reports of the detection of *L. welshimeri* from food samples have been documented in Egypt [52], Turkey [53], and Greece [54].

Regarding the variables for the detection of *L. monocytogenes* in the current study, the prevalence of *L. monocytogenes* varied significantly across the district locations of the retail outlets, a finding in agreement with published studies in South Africa [10] and Bangladesh [55]. However, Ristori et al. [56] did not detect any association between the presence of *L. monocytogenes* in meat products and the geographical regions in Brazil. The disparity in the types of outlets, hygienic practices at the outlets, and the degree of contamination by *Listeria* spp. may account for the differences across regions.

Unsurprisingly, *L. monocytogenes* was detected at the highest frequency (13.2%) in chilled beef and beef products compared with those kept at room temperature and frozen temperatures. The pathogen can survive and multiply at chilling or refrigeration temperatures, which occur at the retail level [57] and during transport. This may affect the number and detection frequency for *Listeria*.

Interestingly, the prevalence of *L. monocytogenes* varied significantly among beef and beef products. This may be attributed to preparation, treatments, or methods of handling, such as brisket to raw spiced beef (“biltong”), raw beef chopped with a knife or grinder (minced meat) to pre-cooked (cold beef/delis). These variable preparation methods and hygienic practices can potentially increase or decrease contamination by *L. monocytogenes* [58].

In our study, *L. monocytogenes* contaminated 14.5% of minced beef samples, which is higher than the prevalence of 1% reported in minced meat in Switzerland [59] and 12.2% in Japan [60]. However, considerably higher frequencies of contamination of minced meat and products by *L. monocytogenes* have been documented in Ireland, 29% [61], Belgium, 42.1% [62], and Brazil, 59.4% [56]. Minced beef and beef products are known to be contaminated by pathogens, primarily due to the preparation methods [56]. Furthermore, minced meat-borne listeriosis outbreaks have been documented [63].

The detection of *L. monocytogenes* in 6.9% of the RTE products is also a food safety concern since RTE beef products have been associated with human listeriosis globally [6]. However, although *L. monocytogenes* was detected in various brands of “biltong” sampled at several sources (home industries, butcheries, supermarkets), the combination of the curing (pH range: 4.81–5.83) and drying (*a*_*w*_ 0.65–0.68) of the product inhibited the survival or growth of the pathogen after marination and within 96 h post-drying [28, 30, 31]. These findings were supported by the report of Gavai et al. [33] that the low internal *a*_*w*_ (<0.85) achieved in assessing “biltong” produced by the standard process in the USA failed to support the survival of *L. monocytogenes.* This is imperative considering that poor sanitary practices exist in the preparation of “biltong,” particularly during production at homes, farms, and butcheries in South Africa that facilitate product contamination. These findings suggest that there is a need to standardize the output of “biltong” in South Africa to assess the potential for moist and dry “biltong” to support the contamination and subsequent proliferation of *L. monocytogenes* at critical control points, specifically during curing (types and concentration of agents) and the temperature and length of drying the product. These factors can potentially influence the contamination, survival, and multiplication of *L. monocytogenes* in “biltong.” For example, all “biltong” samples tested in Botswana were negative for *L. monocytogenes,* as reported by Matsheka et al. [64]. The authors attributed the absence of the pathogen compared to the reported contamination of “biltong” produced in South Africa to the fact that unlike in South Africa, the processing of “biltong” does not involve soaking of meat in cider; the spices are applied directly onto the fresh meat before solar drying in the open air. Furthermore, the mean *a*_*w*_ of the product is 0.51 compared to the mean *a*_*w*_ of 0.65–0.68 for the product in South Africa [28].

It cannot be over-emphasized that “Polony,” another popularly consumed RTE in the country, was responsible for the largest outbreak of human listeriosis [23]. Studies elsewhere have documented a lower prevalence of *L. monocytogenes* in RTE beef products, such as the reported 0.3% [65] and 3.7% to 5.1% in Ethiopia [66] than in our study. However, a higher prevalence of *L. monocytogenes* (44.3%) in RTE beef products was reported in Trinidad and Tobago [67]. The possibility of “polony” still serving as a vehicle and promoter of growth for *L. monocytogenes* in consumers cannot be ignored. The presence of *L. monocytogenes* could be attributed to the contamination of “polony” before and during production and distribution and within the retail environments of this commodity due to the ubiquitous presence of this foodborne pathogen [68]. *Listeria monocytogenes* contaminated 16.7% of our study's “polony” samples.

It is of epidemiological and clinical relevance that of the four distinct serogroups of *L. monocytogenes* reported by Doumith et al. [41], three (4b-4d-4e, 1/2a-3a, and 1/2c-3c) were detected at significantly different frequencies among the *L. monocytogenes* in our study. The relevance is primarily because these serogroups contain seven serotypes (1/2a, 1/2c, 3a, 3c, 4b, 4d, and 4e), of which serotypes 4b, 1/2a, and 1/2c are most frequently reported in animals and humans [69]. It has been reported that diversity in the serotypes and serogroups of *L. monocytogenes* could be attributable to the ecological niche of serotypes [70] and laboratory techniques [71]. It has also been documented that of the 14 *L. monocytogenes* serotypes identified to date, four (1/2a, 1/2b, 1/2c, and 4b) are frequently associated with human listeriosis, with serotypes 4b and 1/2a involved in almost all outbreaks [6, 44, 72, 73].

Of potential virulence and pathogenicity importance is the detection that all (100%) of the isolates of *L. monocytogenes* assessed in the current study were positive for seven of the virulence-associated genes, which included the genes encoding specific virulence factors, specifically, internalins (*inlA, inlB, inlC, inlJ*), hemolysin (*hlyA)*, phospholipase (*plcA*), and actin polymerization (*actA*) in 97.3% of the isolates. Similar findings were reported in a nationwide study on meat and meat products [10] and isolates recovered from cattle farms and cattle abattoirs [10, 74]. However, diversity has been reported in the detection of the virulence genes in *L. monocytogenes* recovered from meat and meat products in the literature, such as *hlyA, prfA,* and *inlA* in Chile [75], *inlC,* and *inlJ* in China [76], and *hlyA*, *actA*, *inlA, inlB, inlC, inlJ*, *prfA*, *plcA*, and *iap* in Turkey and Norway [77, 78]. The roles played by these virulence genes in the pathogenesis of clinical listeriosis following the consumption of *Listeria*-contaminated meat products, particularly RTE foods, are well documented in the literature [76, 79, 80]. The ability of *L. monocytogenes* to cause listeriosis is known to be multifaceted. It has been mainly attributed to six virulence genes, *prfA*, *plcA*, *hly*, *mpl*, *actA*, and *plcB,* which are located in the *PrfA*-dependent virulent gene cluster known as *LIPI-*1 [81, 82], its dependence on genomic islands and, *Listeria* pathogenicity islands, namely, LIPI-1, LIPI-2, LIPI-3, and LIPI-4, and internalin (*inl*) genes, as reported by Gilmour et al. [83] and Wagner et al. [84].

Although the present study determined the frequency of pathogenic serogroups and virulence genes among the isolates of *L. monocytogenes,* a limitation is that the study design did not quantify the number of *L. monocytogenes* per g of beef and beef products. This is because the number of *L. monocytogenes* per g of beef products is essential to assess the risk of listeriosis posed to consumers of contaminated products. Nonetheless, the potential health risk of listeriosis posed to consumers of beef and beef products cannot be ignored, considering that 9.3% were contaminated by pathogenic serogroups, particularly 4b-4d-4e and 1/2a-3a, and were all carriers of virulence genes. Also of health concern is our finding that this pathogen also contaminated 16.7% of the “polony” samples processed. This is because the product was responsible for the country's recent outbreak of human listeriosis, to which Gauteng province contributed 57.93% of the cases nationwide. It is pertinent to mention that there are currently no standards (quantitative or qualitative) for *L. monocytogenes* in foods in South Africa.

## 5. Conclusions

In conclusion, detecting pathogenic serogroups of *L. monocytogenes* in 9.3% of beef and beef products, particularly in RTE foods (6.9%), should be a concern for consumers of these products. This is important because the country recently experienced a large outbreak of human listeriosis, with 59% of cases linked to the consumption of “polony” and RTE beef products. The fact that *L. monocytogenes* was detected in some of these RTE foods in the province is indicative that food safety concerns regarding exposure to the pathogen still exist. However, to better assess the risk of listeriosis posed to consumers of contaminated food products, it is essential to determine the load of *L. monocytogenes* per g of the product before making appropriate recommendations. Thereafter, the WHO/FAO and FDA listeriosis policy described as a “zero tolerance” where a limit of <100 *L. monocytogenes* cells/g at the point of consumption is acceptable can be adopted. The detection of L. *innocua,* a nonpathogen, at a higher frequency than *L. monocytogenes* in all the types of beef and beef products tested may have food safety relevance because it shares similar food niches with *L. monocytogenes* and thus may be predictive of the presence of the pathogen in foods. Overall, it is imperative to reduce or eliminate the contamination of beef and beef products by pathogens such as *L. monocytogenes* through good sanitary practices at slaughterhouses or abattoirs, during transportation, and at retail outlets. Finally, there is a need to conduct a whole genome sequencing and bioinformatics analysis of the isolates of *L. monocytogenes* using their sequence types (ST) to construct the phylogeny to elucidate the clustering or genetic relatedness of the isolates from various sources and sample types.

## Figures and Tables

**Table 1:** Prevalence of *L. monocytogenes*, *L. innocua,* and *L. welshimeri* in beef and beef products, and univariate analysis of associated factors.

Variable	Level	No. of samples tested	No. (%) of samples positive for:		
			*L. monocytogenes* ^1^	*L. innocua* ^2^	*L. welshineri* ^3^
Region	Pretoria North	80	10 (12.5)	10 (12.5)	2 (2.5)
	Pretoria East	90	10 (11.1)	15 (16.7)	0 (0.0)
	Pretoria West	76	9 (11.8)	12 (15.8)	2 (2.6)
	Pretoria South	79	1 (1.3)	18 (22.8)	3 (3.8)
	Pretoria Central	75	7 (9.3)	10 (13.3)	3 (4.0)
	*p* value		0.036	0.434	0.461

Type of outlet^4^	Supermarket chain	115	12 (10.4)	15 (13.0)	1 (0.9)
	Large	108	7 (6.5)	8 (7.4)	4 (3.7)
	Medium	107	7 (6.5)	18 (16.8)	3 (2.8)
	Small	70	11 (15.7)	13 (18.6)	2 (2.9)
	*p* value		0.133	0.726	0.598

Type of product	Beef burger	40	0 (0.0)	5 (12.5)	0 (0.0)
	Biltong	55	2 (3.6)	7 (12.7)	1 (1.8)
	Minced beef	110	16 (14.5)	21 (19.1)	2 (1.8)
	Brisket	120	12 (10.0)	22 (18.3)	7 (5.8)
	Cold beef (deli)	75	7 (9.3)	10 (13.3)	0 (0.0)
	*p* value		0.032	0.674	0.112

Storage temperature	Non-chilled^5^	55	3 (5.5)	6 (10.9)	1 (1.8)
	Chilled	220	29 (13.2)	51 (23.2)	8 (3.6)
	Frozen	125	5 (4.0)	8 (6.4)	1 (0.8)
	*p* value		0.011	<0.001	0.252

Status of the product	RTE^6^	130	9 (6.9)	15 (11.5)	1 (0.8)
	Raw	270	28 (10.4)	50 (18.5)	9 (3.3)
	*p* value		0.265	0.076	0.177

^1^The overall prevalence of *L. monocytogenes* was 9.3% (37/400); the ^2^overall prevalence of *L. innocua* was 16.3% (65/400), and the ^3^overall prevalence of *L. welshimeri* was 2.5% (10/400); ^4^Supermarket chain: Over one outlet, Large: 6 or more Cashiers, Medium: 3–5 cashiers, and Small: 1-2 cashiers, ^5^At room temperature, ^6^RTE: Ready-to-eat; deli beef: (“Polony” and Vienna).

**Table 2:** Frequency of detection of *L. monocytogenes* serogroups by the region, size of retail outlets, and type of beef and beef products.

Variable	No. of isolates of *L. monocytogenes*^1^	No. (%) of isolates belonging to serogroup		
		1/2a-3a	1/2c-3c	4b-4d-4e
*Region*				
Pretoria North	10	2 (20.0)	1 (10.0)	7 (70.0)
Pretoria East	10	6 (60.0)	1 (10.0)	3 (30.0)
Pretoria West	9	7 (77.8)	0 (0.0)	2 (22.2)
Pretoria South	1	0 (0.0)	0 (0.0)	1 (100.0)
Pretoria Central	7	1 (14.3)	0 (0.0)	6 (85.7)
*p* value		0.183	1	0.023

*Type of retail outlet* ^2^				
Supermarket chain	12	6 (50.0)	0 (0.0)	6 (50.0)
Large	7	2 (28.6)	2 (28.6)	3 (42.9)
Medium	7	3 (42.9)	0 (0.0)	4 (57.1)
Small	11	5 (45.5)	0 (0.0)	6 (54.5)
*p* value		0.003	0.462	0.950

*Type of beef and beef products*				
“Biltong”	2	0 (0.0)	0 (0.0)	2 (100.0)
Minced beef	16	6 (37.5)	1 (6.3)	9 (56.3)
Brisket	12	7 (58.3)	1 (8.3)	4 (33.3)
Cold beef	7	3 (42.9)	0 (0.0)	4 (57.1)
*p* value		0.541	0.832	0.428

^1^Of a total of 37 isolates of L. monocytogenes. ^2^Supermarket chain: Over 1 outlet, large: 6 or more cashiers, medium: 3–5 cashiers, and small: 1–2 cashiers.

## Data Availability

All the data are contained within the article.

## Supplementary Materials

Supplementary Materials Supplementary information includes the number of samples collected across 48 retail outlets and the frequency of detection of *L. monocytogenes*, *L. innocua,* and *L. welshimeri* from beef and beef products sampled from retail outlets, the strategy used for sample collection from classes of retail outlets, the primers used for mPCR speciation, serogrouping, and virulence gene detection *in L. monocytogenes* isolates.
