# Supplementary material for: Detection of Pathogenic Serogroups and Virulence Genes in Listeria monocytogenes Strains Isolated from Beef and Beef Products Retailed in Gauteng Province, South Africa, Using Phenotypic and Polymerase Chain Reaction (PCR)-Based Methods
Source: Int J Microbiol. 2024 Mar 13;2024:8891963. doi: 10.1155/2024/8891963 (PMC10954364; doi:10.1155/2024/8891963)
Supplement: Supplementary Materials — Supplementary information includes the number of samples collected across 48 retail outlets and the frequency of detection of L. monocytogenes, L. innocua, and L. welshimeri from beef and beef products sampled from retail outlets, the strategy used for sample collection from classes of retail outlets, the primers used for mPCR speciation, serogrouping, and virulence gene detection in L. monocytogenes isolates. [file 8891963.f1.docx]

**SUPPLEMENTARY DATA**

**Supplementary Data: Table S1-S5**

**Supplementary data, Table S1: Number of samples collected across 48 retail outlets and the frequency of detection of *L. monocytogenes*, *L. innocua,* and *L. welshimeri* from beef and beef products sampled from retail outlets in Gauteng Province, South Africa**

| **Outlets** | **No. of samples collected** | **No. (%) positive for L. *monocytogenes*** | **No, (%) positive for *L. innocua*** | **No. (%) positive for *L. welshimeri*** | **Total No, (%) positive for Listeria spp.** |
| --- | --- | --- | --- | --- | --- |
| 1 | 8 | 2 (25.0) | 0 (0.0) | 1 (12.5) | 3 (37.5) |
| 2 | 8 | 0 (0.0) | 2 (25.0) | 0 (0.0) | 2 (25.0) |
| 3 | 8 | 1 (12.5) | 1 (12.5) | 0 (0.0) | 2 (25.0) |
| 4 | 8 | 0 (0.0) | 3 (37.5) | 0 (0.0) | 3 (37.5) |
| 5 | 8 | 0 (0.0) | 2 (25.0) | 0 (0.0) | 2 (25.0) |
| 6 | 8 | 0 (0.0) | 0 (0.0) | 2 (25.0) | 2 (25.0) |
| 7 | 8 | 2 (25.0) | 1 (12.5) | 0 (0.0) | 3 (37.5) |
| 8 | 8 | 1 (12.5) | 1 (12.5) | 0 (0.0) | 2 (25.0) |
| 9 | 8 | 1 (12.5) | 0 (0.0) | 0 (0.0) | 1 (12.5) |
| 10 | 8 | 1 (12.5) | 0 (0.0) | 0 (0.0) | 1 (12.5) |
| 11 | 9 | 0 (0.0) | 3 (33.3) | 0 (0.0) | 3 (33.3) |
| 12 | 9 | 0 (0.0) | 2 (22.2) | 0 (0.0) | 2 (22.2) |
| 13 | 9 | 0 (0.0) | 1 (11.1) | 0 (0.0) | 1 (11.1) |
| 14 | 9 | 2 (22.2) | 0 (0.0) | 0 (0.0) | 2 (22.2) |
| 15 | 9 | 1 (11.1) | 0 (0.0) | 0 (0.0) | 1 (11.1) |
| 16 | 9 | 1 (11.1) | 3 (33.3) | 0 (0.0) | 4 (44.4) |
| 17 | 8 | 0 (0.0) | 2 (25.0) | 0 (0.0) | 2 (25.0) |
| 18 | 8 | 0 (0.0) | 3 (37.5) | 0 (0.0) | 3 (37.5) |
| 19 | 8 | 2 (25.0) | 1 (12.5) | 0 (0.0) | 3 (37.5) |
| 20 | 8 | 0 (0.0) | 0 (0.0) | 0 (0.0) | 0 (0.0) |
| 21 | 8 | 0 (0.0) | 2 (25.0) | 0 (0.0) | 2 (25.0) |
| 22 | 8 | 2 (25.0) | 1 (12.5) | 0 (0.0) | 3 (37.5) |
| 23 | 9 | 3 (33.3) | 0 (0.0) | 0 (0.0) | 3 (33.3) |
| 24 | 9 | 1 (11.1) | 3 (33.3) | 0 (0.0) | 4 (44.4) |
| 25 | 9 | 3 (33.3) | 1 (11.1) | 1 (11.1) | 5 (55.6) |
| 26 | 8 | 0 (0.0) | 1 (12.5) | 0 (0.0) | 1 (12.5) |
| 27 | 8 | 1 (12.5) | 1 (12.5) | 0 (0.0) | 2 (25.0) |
| 28 | 8 | 1 (12.5) | 1 (12.5) | 0 (0.0) | 2 (25.0) |
| 29 | 8 | 0 (0.0) | 0 (0.0) | 0 (0.0) | 0 (0.0) |
| 30 | 8 | 0 (0.0) | 3 (37.5) | 1 (12.5) | 4 (50.0) |
| 31 | 8 | 1 (12.5) | 2 (25.0) | 0 (0.0) | 3 (37.5) |
| 32 | 9 | 2 (22.2) | 1 (11.1) | 1 (11.1) | 4 (44.4) |
| 33 | 9 | 0 (0.0) | 4 (44.4) | 0 (0.0) | 4 (44.4) |
| 34 | 9 | 2 (22.2) | 1 (11.1) | 0 (0.0) | 3 (33.3) |
| 35 | 8 | 0 (0.0) | 2 (25.0) | 0 (0.0) | 2 (25.0) |
| 36 | 8 | 1 (12.5) | 1 (12.5) | 1 (12.5) | 3 (37.5) |
| 37 | 8 | 1 (12.5) | 0 (0.0) | 0 (0.0) | 1 (12.5) |
| 38 | 8 | 0 (0.0) | 2 (25.0) | 0 (0.0) | 2 (25.0) |
| 39 | 9 | 0 (0.0) | 3 (33.3) | 0 (0.0) | 3 (33.3) |
| 40 | 9 | 1 (11.1) | 0 (0.0) | 2 (22.2) | 3 (33.3) |
| 41 | 9 | 0 (0.0) | 1 (11.1) | 0 (0.0) | 1 (11.1) |
| 42 | 9 | 1 (11.1) | 3 (33.3) | 0 (0.0) | 4 (44.4) |
| 43 | 8 | 1 (12.5) | 1 (12.5) | 0 (0.0) | 2 (25.0) |
| 44 | 8 | 0 (0.0) | 2 (25.0) | 0 (0.0) | 2 (25.0) |
| 45 | 8 | 0 (0.0) | 1 (12.5) | 0 (0.0) | 1 (12.5) |
| 46 | 8 | 0 (0.0) | 0 (0.0) | 1 (12.5) | 1 (12.5) |
| 47 | 8 | 1 (12.5) | 2 (25.0) | 0 (0.0) | 3 (37.5) |
| 48 | 8 | 1 (12.5) | 1 (12.5) | 0 (0.0) | 2 (25.0) |

**Supplementary data, Table S2. The strategy used for sample collection from classes of retail outlets in Gauteng province**

|  |  |  | **Number of samples collected:** | | | | | | | | | | | | | | |
| --- | --- | --- | --- | --- | --- | --- | --- | --- | --- | --- | --- | --- | --- | --- | --- | --- | --- |
| **Classification of** |  | **Maximum No. of**  **samples to collect/outlet** | **Brisket/Raw beef** | | | | **Boerewors/Minced** | | **Cold meat** | | | **Biltong** | | **Beef** | **Beef** | **Total No. of :** | |
| **Supermarket^s1^** |  |  | **Beef steak** | **Liver** | **Tripe** | **Chunk** | **Sausages** | **Meat** | **Beef Polony** | **Russian Polony** | **Vienna** | **Moist** | **Dry** | **Patties** | **Burgers** | **Outlets** | **Samples** |
|  |  |  |  |  |  |  |  |  |  |  |  |  |  |  |  |  |  |
| Chain |  | 12 | 12 | 12 | 12 | 12 | 12 | 12 | 13 | 13 | 13 | 12 | 12 | 12 | 13 | 30 | 160 |
|  |  |  |  |  |  |  |  |  |  |  |  |  |  |  |  |  |  |
| Large |  | 8—10 | 10 | 10 | 10 | 10 | 10 | 10 | 10 | 10 | 10 | 9 | 9 | 10 | 10 | 10 | 128 |
|  |  |  |  |  |  |  |  |  |  |  |  |  |  |  |  |  |  |
| Medium |  | 4—6 | 6 | 7 | 6 | 6 | 6 | 6 | 7 | 6 | 6 | 6 | 6 | 6 | 6 | 6 | 80 |
|  |  |  |  |  |  |  |  |  |  |  |  |  |  |  |  |  |  |
| Small |  | 1—2 | 2 | 3 | 3 | 2 | 3 | 3 | 3 | 2 | 2 | 2 | 2 | 2 | 3 | 2 | 32 |

**Supplementary data, Table S3: Primers used for mPCR speciation in this study [40]**

| **Species** | **Gene** | **Primer** | **Sequences (5^i^-3^i^)** | **PCR Product Size (bp)** |
| --- | --- | --- | --- | --- |
| *Listeria* genus | *Prs* | *prs-F* | GCTGAAGAGATTGCGAAAGAAG | 370 |
|  |  | *prs-R* | CAAAGAAACCTTGGATTTGCGG |  |
| *L.* *grayi* | *Oxidoreductase* | *JOgrayi-F* | GCGGATAAAGGTGTTCGGGTCAA | 201 |
|  |  | *JOgrayi-R* | ATTTGCTATCGTCCGAGGCTAGG |  |
| *L.* *innocua* | *lin0464* | *lin0464-F* | CGCATTTATCGCCAAAACTC | 749 |
|  |  | *lin0464-R* | TCGTGACATAGACGCGATTG |  |
| *L. ivanovii* | *namA* | *liv22-228-F* | CGAATTCCTTATTCACTTGAGC | 463 |
|  |  | *liv22-228-R* | GGTGCTGCGAACTTAACTCA |  |
| *L. monocytogenes* | *Imo1030* | *lmo1030-F* | GCTTGTATTCACTTGGATTTGTCTGG | 509 |
|  |  | *lmo1030-R* | ACCATCCGCATATCTCAGCCAACT |  |
| *L.* *seeligeri* | *lmo033* | *lseelin-F* | GTACCTGCTGGGAGTACATA | 673 |
|  |  | *lseelin-R* | CTGTCTCCATATCCGTACAG |  |
| *L.* *welshimeri* | *scrA* | *lwe1801-F* | CGTGGCACAATAGCAATCTG |  |
|  |  | *lwe1801-R* | GACATGCCTGCTGAACTAGA | 281 |

**Supplementary data, Table S4: Primers used for mPCR to serogroup *L. monocytogenes* in the study [41]**

| PCR assay | Target Gene | Product Size (bp) | Primer Sequences (5′‐3’) |
| --- | --- | --- | --- |
| mPCR | *ORF2110* | 597 | *ORF2110*‐F:  *AGTGGACAATTGATTGGTGAA* |
|  |  |  | *ORF2110*‐R: *CATCCATCCCTTACTTTGGAC* |
|  | *ORF2819* | 471 | *ORF2819*‐F: *AGCAAAATGCCAAAACTCGT* |
|  |  |  | *ORF2819*‐R: *CATCACTAAAGCCTCCCATTG* |
|  | *Imo1118* | 906 | *lmo1118*‐F: *AGGGGTCTTAAATCCTGGAA* |
|  |  |  | *Imo1118*‐R: *CGGCTTGTTCGGCATACTTA* |
|  | *Imo0737* | 691 | *lmo0737*‐F: *AGGGCTTCAAGGACTTACCC* |
|  |  |  | *lmo0737*‐R: *ACGATTTCTGCTTGCCATTC* |
|  | *Prs* | 370 | *prs*‐ F: GCTGAAGAGATTGCGAAAGAAG |
|  |  |  | *prs‐****R****: CAAAGAAACCTTGGATTTGCGG* |

**Supplementary data, Table S5: Primer sequences, PCR preparation, and PCR condition used for virulence gene detection in this study**

| **PCR** | **Target** | **Product** |  |  |
| --- | --- | --- | --- | --- |
| **Assay** | **Gene** | **size (bp)** | **Primer sequences (5^’^ 3’)** | **References** |
| mPCR1 | *plcA* | 1484 | *plcA*‐F: CTGCTTGAGCGTTCATGTCTCATCCCCC | Rawool et al. [42] |
|  |  |  | *plcA*‐R: CATGGGTTTCACTCTCCTTCTAC |  |
|  | *hlyA* | 456 | *hly*A*‐*F: GTTAATGAACCTACAAGACCTTCC | Rawool et al. [42] |
|  |  |  | *hly*A*‐*R: ACCGTTCTCCACCATTCCCA |  |
|  | *actA* | 839 | *actA*‐F: CGCCGCGGAAATTAAAAAAAGA | Rawool et al. [42] |
|  |  |  | *actA*‐R: ACGAAGGAACCGGGCTGCTAG |  |
|  | *Iap* | 131 | *iap*‐F: ACAAGCTGCACCTGTTGCAG | Rawool et al. [42] |
|  |  |  | *iap*‐R: TGACAGCGTGTGTAGTAGCA |  |
| mPCR2 | *inlA* | 800 | *inlA*‐F: ACGAGTAACGGGACAAATGC | Liu et al. [43] |
|  |  |  | *inlA*‐R: CCCGACAGTGGTGCTAGATT |  |
|  | *inlB* | 367 | *inlB‐F:* GATATTGTGCCACTTTCAGGTT | Liu et al. [43] |
|  |  |  | *inlB‐R:* CCTCTTTCAGTGGTTGGGTT |  |
|  | *inlC* | 517 | *inlC*‐F: AATTCCCACAGGACACAACC | Liu et al. [43] |
|  |  |  | i*nlC‐R:*CGGGAATGCAATTTTTCACTA |  |
|  | *inlJ* | 238 | *inlJ*‐F: TGTAACCCCGCTTACACAGTT | Liu et al. [43] |
|  |  |  | *inlJ‐R:* AGCGGCTTGGCAGTCTAATA |  |
|  |  |  |  |  |
